# Supplementary material for: Validation of prognostic indices for short term mortality in an incident dialysis population of older adults >75
Source: PLoS One. 2021 Jan 20;16(1):e0244081. doi: 10.1371/journal.pone.0244081 (PMC7816982; doi:10.1371/journal.pone.0244081)
Supplement: S1 Table — (DOCX) [file pone.0244081.s002.docx]

**S1 Table. Variables used to construct Barthel score**

| **Question** | **Points assigned** |
| --- | --- |
| Do you have difficulty eating by yourself? | 2 if yes, 0 if no |
| Do you have difficulty dressing by yourself? | 2 if yes, 0 if no |
| Do you have difficulty using the toilet by yourself? | 2 if yes, 0 if no |
| Do you have difficulty bathing by yourself? | 2 if yes, 0 if no |
| Do you have difficulty walking by yourself? | 2 if yes, 0 if no |
| Do you have difficulty housekeeping by yourself? | 2 if yes, 0 if no |
| Do you have difficulty getting in and out of bed by yourself? | 2 if yes, 0 if no |
| Do you depend on any assistive devices (e.g. cane, wheelchair, braces, walker, etc.) or assistance from other people to perform activities important to you in your daily life? | 2 if yes, 0 if no |
| Can you climb two flights of stairs without stopping to rest? | 2 if no,  1 if yes with difficulty,  0 if yes, with no difficulty |

*Barthel score calculated as: total score / 18 *100
