# Supplementary material for: Validation of prognostic indices for short term mortality in an incident dialysis population of older adults >75
Source: PLoS One. 2021 Jan 20;16(1):e0244081. doi: 10.1371/journal.pone.0244081 (PMC7816982; doi:10.1371/journal.pone.0244081)
Supplement: S2 Table — (DOCX) [file pone.0244081.s003.docx]

**S2 Table. Variables used in current study to construct risk scores**

| **Variable** | NCI | Wick | REIN | Updated REIN | Foley | Thamer |
| --- | --- | --- | --- | --- | --- | --- |
| Age |  | X |  | X | X | X |
| Gender |  | X^1^ |  | X |  |  |
| Race |  | X^1^ |  |  |  |  |
| Functional status |  |  | X |  |  | X |
| Behavioral disorder |  |  | X | X |  |  |
| CHF | X | X | X | X | X | X |
| Sepsis |  |  |  |  | X |  |
| CAD/ASHD | X |  |  |  | X |  |
| CVA/TIA | X |  |  |  |  |  |
| PVD | X |  | X | X | X |  |
| COPD | X |  |  |  |  |  |
| Liver Disease | X |  |  |  |  |  |
| Dysrhythmia/AFIB | X | X | X | X | X |  |
| Cancer (any) | X | X | X | X | X | X |
| Lymphoma |  | X |  |  |  |  |
| Diabetes | X |  | X |  |  |  |
| Hypertension | X |  |  |  |  |  |
| GI Bleed | X |  |  |  |  |  |
| On ventilator/ in coma |  |  |  |  | X |  |
| BMI, kg/m^2^ |  |  | X |  |  |  |
| Serum albumin, g/dl |  |  |  | X |  | X |
| Creatinine |  | X^1^ |  |  |  |  |
| eGFR (ml/min/1.73 m^2^) |  | X |  |  |  |  |
| Barthels score |  |  |  | X |  |  |
| In the past year: >1 hospital visit or hospitalized for >1 month |  |  |  |  |  | X |
| Unplanned dialysis initiation |  |  | X |  |  |  |
| Mortality | X | X | X | X | X | X |

1. Used in eGFR calculation, not in score construction
